# Chloride:Sodium Ratio May Accurately Predict Corrected Chloride Disorders and the Presence of Unmeasured Anions in Dogs and Cats

**DOI:** 10.3389/fvets.2017.00122

**Published:** 2017-08-02

**Authors:** Robert Goggs, Marc Myers, Sage De Rosa, Erik Zager, Daniel J. Fletcher

**Affiliations:** ^1^Department of Clinical Sciences, College of Veterinary Medicine, Cornell University, Ithaca, NY, United States; ^2^MedVet Columbus, Worthington, OH, United States; ^3^Department of Clinical Studies, University of Pennsylvania School of Veterinary Medicine, Philadelphia, PA, United States

**Keywords:** strong ion gap, acid–base, metabolic acidosis, canine, feline

## Abstract

Disorders of chloride and mixed acid–base disturbances are common in veterinary emergency medicine. Rapid identification of these alterations and the presence of unmeasured anions aid prompt patient assessment and management. This study aimed to determine in dogs and cats if site-specific reference values for [Cl^−^]:[Na^+^] ratio and [Na^+^] − [Cl^−^] difference accurately identify corrected chloride abnormalities and to evaluate the predictive ability of the [Cl^−^]:[Na^+^] ratio for the identification of unmeasured anions. A database containing 33,117 canine, and 7,604 feline blood gas and electrolyte profiles was generated. Institution reference intervals were used to calculate site-specific reference values for the [Cl^−^]:[Na^+^] ratio and the [Na^+^] − [Cl^−^] difference. Contingency tables were used to assess the ability of these values to correctly identify corrected chloride disorders. Unmeasured anions were estimated by calculating strong ion gap (SIG). Continuous variables were compared using the Mann–Whitney *U* test. Correlations between continuous variables were assessed using Spearman’s rho (*r*_s_). In dogs, site-specific reference values for the [Cl^−^]:[Na^+^] ratio correctly identified 94.6% of profiles as hyper-, normo-, or hypochloremic. For dogs with normal sodium concentrations, site-specific reference values for the [Na^+^] − [Cl^−^] difference correctly identified 97.0% of profiles. In dogs with metabolic acidosis (base deficit > 4.0), [Cl^−^]:[Na^+^] ratio and SIG were moderately but significantly negatively correlated (*r*_s_ −0.592, *P* < 0.0001). SIG was significantly greater in dogs with metabolic acidosis and hypochloremia compared to those without hypochloremia (*P* < 0.0001). In cats, site-specific reference values for the [Cl^−^]:[Na^+^] ratio correctly identified 93.3% of profiles as hyper-, normo-, or hypochloremic, while site-specific reference values for [Na^+^] − [Cl^−^] difference correctly identified 95.1% of profiles. In cats with metabolic acidosis [Cl^−^]:[Na^+^] ratio and SIG were moderately significantly negatively correlated (*r*_s_ −0.730, *P* < 0.0001). SIG was significantly greater in cats with metabolic acidosis and hypochloremia compared to those without hypochloremia (*P* < 0.0001). Site-specific values for [Cl^−^]:[Na^+^] ratio and [Na^+^] − [Cl^−^] difference accurately identify corrected chloride disorders in both dogs and cats and may aid identification of the presence of unmeasured anions.

## Introduction

Chloride is the principal anion in the extracellular fluid (ECF) and thus its regulation is crucial for maintenance of osmolality and for acid–base balance (1). Metabolic acid–base disorders are common in veterinary emergency and critical care medicine and point of care measurement of electrolytes and acid–base status are integral to the diagnosis and management of emergency patients (2, 3). The acid–base status of plasma (and hence the ECF) is affected by alterations in alveolar ventilation changing PaCO_2_, through manipulation of the plasma strong ion difference (SID) by the kidneys and secondary to alterations in the concentrations of weak acids, as indicated by *A*_tot_ (4, 5). The kidneys regulate SID by differential reabsorption of sodium and chloride ions in the renal tubules. Disorders of plasma chloride concentration are strongly associated with acid–base disturbances. A decrease in plasma chloride increases SID, causing a hypochloremic alkalosis, while an increase in plasma chloride decreases SID, causing a hyperchloremic acidosis (1). Identification of such alterations can be clinically valuable to increase the index of suspicion for gastrointestinal obstruction (6), or the presence of unmeasured anions.

In addition to disorders that cause gain or loss of chloride, changes in plasma chloride concentration can also result from changes in water balance. When changes in water balance affect measured chloride concentrations, the measured sodium concentration also changes (7). Correct identification of a true chloride change occurring independent of changes in water balance requires calculation of the corrected chloride, by taking into account the corresponding changes in sodium concentration:

(1) $${\left[{\text{Cl}}^{-}\right]}_{\text{Corrected}}=({\left[{\text{Na}}^{+}\right]}_{\text{Normal}}/{\left[{\text{Na}}^{+}\right]}_{\text{Measured}})\times {\left[{\text{Cl}}^{-}\right]}_{\text{Measured}}$$

Typically, the mid-point of the sodium reference interval (RI) is used to approximate normal sodium for this calculation. The corrected chloride value is then compared against the institution RIs for chloride to determine if an independent chloride disorder exists. An alternative method for rapid identification of the presence of a corrected chloride disorder is the calculation of the chloride:sodium ratio ([Cl^−^]:[Na^+^] ratio) (8).

A high [Cl^−^]:[Na^+^] ratio suggests a gain of chloride relative to sodium and a hyperchloremic metabolic acidosis independent of free-water changes, while a low chloride to sodium ratio suggests a loss of chloride relative to sodium and a hypochloremic metabolic alkalosis. Reference values for the chloride to sodium ratio have not been established for dogs and cats. Anecdotally, it has been suggested that values >0.78 in dogs and >0.80 in cats are associated with hyperchloremic metabolic acidosis, while values <0.72 in dogs and <0.74 in cats are associated with hypochloremic alkalosis (9). Since the corrected chloride calculation and the chloride to sodium ratio calculation incorporate the same values, the two calculations are mathematically linked. Thus, it is possible for every institution to calculate site-specific reference values for the [Cl^−^]:[Na^+^] ratio as follows:

(2) $$\text{Cl:Na ratio lower bound}=\left[{\text{Cl}}^{-}\right]\text{RI lower bound}/\left[{\text{Na}}^{+}\right]\text{RI midpoint}$$

(3) $$\text{Cl:Na ratio upper bound}=\left[{\text{Cl}}^{-}\right]\text{RI upper bound}/\left[{\text{Na}}^{+}\right]\text{RI midpoint}$$

When the sodium concentration is normal, the difference between the sodium and chloride concentrations ([Na^+^] − [Cl^−^]) can also be used to identify the presence of a corrected chloride disorder. If sodium is abnormal then using a simple difference will either underestimate or overestimate the influence of free water on chloride levels. Normal values for the sodium-chloride difference have also been previously reported, wherein values >40 mmol/L indicate hypochloremic alkalosis, while values <32 mEq/L are associated with hyperchloremic acidosis, but these values have not been validated to date (9). Following similar logic to the site-specific chloride to sodium ratio calculation, a site-specific RI for the sodium chloride difference may be calculated as:

(4) $$\left[{\text{Na}}^{+}\right]-\left[{\text{Cl}}^{-}\right]\text{difference lower bound}=\left[{\text{Na}}^{+}\right]\text{RI midpoint}-\left[{\text{Cl}}^{-}\right]\text{RI upper bound}$$

(5) $$\left[{\text{Na}}^{+}\right]-\left[{\text{Cl}}^{-}\right]\text{difference upper bound}=\left[{\text{Na}}^{+}\right]\text{RI midpoint}-\left[{\text{Cl}}^{-}\right]\text{RI lower bound}$$

A study in human pediatrics suggested that both the [Cl^−^]:[Na^+^] ratio and the [Na^+^] − [Cl^−^] difference were significantly different in patients with acidosis and unmeasured anions compared to those without unmeasured anions (10). In addition, the [Cl^−^]:[Na^+^] ratio was as discriminating as the anion gap for identification of tissue acidosis and performed better than lactate or base deficit (BD) measures.

The present study therefore aimed to determine in both dogs and cats if site-specific reference values for [Cl^−^]:[Na^+^] ratio and [Na^+^] − [Cl^−^] difference accurately identify corrected chloride abnormalities, and to evaluate the predictive ability of the [Cl^−^]:[Na^+^] ratio for the identification of increased unmeasured anions and mixed acid–base disturbances. We hypothesized that use of site-specific reference values for the [Cl^−^]:[Na^+^] ratio and [Na^+^] − [Cl^−^] difference would identify corrected chloride abnormalities more accurately than the [Cl^−^]:[Na^+^] ratio values suggested by de Morais and Leisewitz (9); that corrected hypochloremia in the presence of a BD correlates with the presence of unmeasured anions and that corrected normochloremia in the presence of a BD correlates with the presence of mixed acid–base disturbances.

## Materials and Methods

### Electrolyte and Metabolite Analyses

Blood gas and electrolyte analyses were conducted with point-of-care analyzers (RapidPoint 405; Siemens, Malvern, PA, USA) equipped with ion-selective electrodes using blood samples heparinized with dry balanced lithium/zinc heparin (Arterial Blood Gas Sampler, Westmed Inc., Tucson, AZ, USA). The point-of-care instrument employed in this study uses an external cartridge-based system to provide quality control (QC). This cartridge contains three separate levels of control material that span clinically relevant ranges. The QC procedure on the analyzer is automated and runs at a predetermined frequency (three times per day). Analysis channels that fail repeated QC are turned off and do not produce patient test results until the problem is rectified by an operator. Day-to-day and month-on-month performance based on tabulated QC data and Levey-Jennings graphs is assessed as required. Local RIs for the blood-gas analyzer were previously generated using heparinized blood samples collected from 20 healthy dogs and 20 healthy cats that were not part of the study population. These animals were considered healthy on the basis of history, physical examination and the results of complete blood count and serum chemistry profiles. Serum chemistry analyses were conducted using an automated chemistry analyzer (Cobas ModP, Roche-Hitachi, Indianapolis, IN, USA). Blood lactate concentrations were measured with a handheld point-of-care lactate meter (Lactate Pro, Arkray, Minneapolis, MN, USA) using heparinized whole blood samples.

### Case Selection and Database Compilation

An electronic database of blood gas and electrolyte analyses conducted at Cornell University Hospital for Animals between May 31, 2007, and January 03, 2015, on patients presented to the emergency room or intensive care unit was searched for results from dogs and cats. The database was visually inspected and manually curated to remove samples from species other than dogs and cats, samples with missing, erroneous or untraceable case numbers, analyses from sample types other than blood (e.g. abdominal fluid) and analyses with missing data. Medical records from dogs with very high measured chloride concentrations were manually checked to identify patients receiving potassium bromide as an anticonvulsant (11). These cases were removed from the dataset to limit the impact of a known interfering substance (12). Provided the datasets were complete, multiple samples from a single animal were kept in the database. Institution computerized medical record systems were then searched for data on patient signalment, presenting complaint, final diagnosis, outcome, hospitalization dates and for data from serum biochemistry analyses. Four separate databases were thereby created containing the electrolyte data, point-of-care analyses, biochemistry analyses and case demographics. A custom script (Visual Basic, Microsoft Visual 328 Studio for Windows; Microsoft, Redmond, WA, USA) was written to search each database via the unique patient identifier and created a final composite database containing the relevant data from each of the separate databases corresponding to the time and date stamp from the blood-gas and electrolyte analyses. The final database was then manually checked for accuracy by cross-referencing the database entries with the parent data sources for a randomized selection of cases, spanning the entire range of case numbers and representing 0.1% of the total case entries. From the parent database, canine and feline cases were segregated for further analyses. For calculation of acid–base variables, only cases where all necessary variables were contemporaneously measured were selected.

### Electrolyte Reference Value Calculations

Canine corrected chloride values were calculated as (148/[Na^+^]_Measured_) × [Cl^−^]_Measured_, while feline corrected chloride values were calculated as (153/[Na^+^]_Measured_) × [Cl^−^]_Measured_. Based on the midpoints (mean values) of the institution RIs for sodium and chloride, site-specific [Cl^−^]:[Na^+^] ratio reference values were calculated to be 0.7432–0.8041 in dogs (110/148 and 119/148) and 0.7647–0.8301 (117/153 and 127/153) in cats. To make these intervals more clinically applicable they were rounded to two decimal places 0.74–0.80 (dogs) and 0.76–0.83 (cats). Site specific [Na^+^] − [Cl^−^] difference RIs were calculated to be 29–38 mmol/L (148–119 and 148–110) in dogs and 26–36 mmol/L (153–127 and 153–117) in cats.

### Acid–Base and Strong Ion Calculations

Unmeasured anions were estimated by calculating the strong ion gap (SIG) as follows (2, 13, 14):

(6) $$\text{SIG}={\text{SID}}_{\text{apparent}}-{\text{SID}}_{\text{effective}}$$

(7) $$\text{SIG}=((\left[{\text{Na}}^{+}\right]+\left[{\text{K}}^{+}\right])-(\left[{\text{Cl}}^{-}\right]+\left[\text{Lactate}\right]))-(\left[{\text{HCO}}_{3}{}^{-}\right]+\text{Alb}.\text{contribution}+\text{Phos}.\text{contribution})$$

where

Alb.contribution=[Albumin](g/dL)×2.8

Phos.contribution=[Phosphate](mg/dL)×0.58.

### Statistical Analyses

Prior to test selection, variables were tested for normality using the D’Agostino Pearson test, and descriptive statistics calculated. Parametric variables are presented as mean ± SD, while non-parametric variables are presented as median (min-max). Non-parametric continuous variables were compared using the Mann–Whitney *U* test and with box and whisker plots. Correlations between non-parametric continuous variables were assessed by calculating Spearman’s rho (*r*_s_). Diagnostic accuracy of the site-specific reference values was calculated as (True Positive + True negative)/(True Positive + False Positive + True Negative + False Negative). Alpha was set at 0.05. Statistical analyses were conducted using commercial software (Prism 7 for Mac OS X; GraphPad Software, La Jolla, CA, USA).

## Results

### Dogs

There were 33,117 complete canine electrolyte profiles available for examination. Based on calculations using equation 1, 20,908 profiles had normal chloride, 6,482 profiles were hypochloremic, and 5,727 profiles showed evidence of hyperchloremia. The previously reported interval for the [Cl^−^]:[Na^+^] ratio in dogs (0.72–0.78) correctly identified 13,731 of the normal profiles, 2,068 of the hypochloremic profiles, and 5,727 of the hyperchloremic profiles for a total accuracy of 65%. The site-specific reference values (0.74–0.80) correctly identified 20,011 of the normal profiles, 5,577 of the hypochloremic profiles were correctly identified, and 5,727 of the hypochloremic profiles were correctly identified, for a total accuracy percentage of 94.56%. Overall a net improvement in accuracy of 29.56% over the previously published RI was attained.

Only profiles with normal sodium concentrations were used to assess the performance of the Na-Cl difference RIs. There were 16,662 profiles with normal sodium concentrations. Based on calculations using equation 1, of the 16,662 profiles that were normonatremic, 11,131 profiles had normal chloride, 3,113 profiles were hypochloremic, and 2,418 profiles showed evidence of hyperchloremia. The previously reported RIs for the Na-Cl difference (32–40) correctly identified 8,355 of the normal profiles, 1,467 of the hypochloremic profiles, and 2,418 of the hyperchloremic profiles for a total accuracy percentage of 73.46%. Using the newly proposed normal ranges for Na-Cl difference evaluation (29.0–38.0), 10,863 of the normal profiles were correctly identified, 2,946 of the hypochloremic profiles were correctly identified, and 2,352 of the hypochloremic profiles were correctly identified, for a total accuracy percentage of 96.99%. Overall a net improvement of 23.53% in accuracy was attained over the previously published RI.

**Figure 1:**
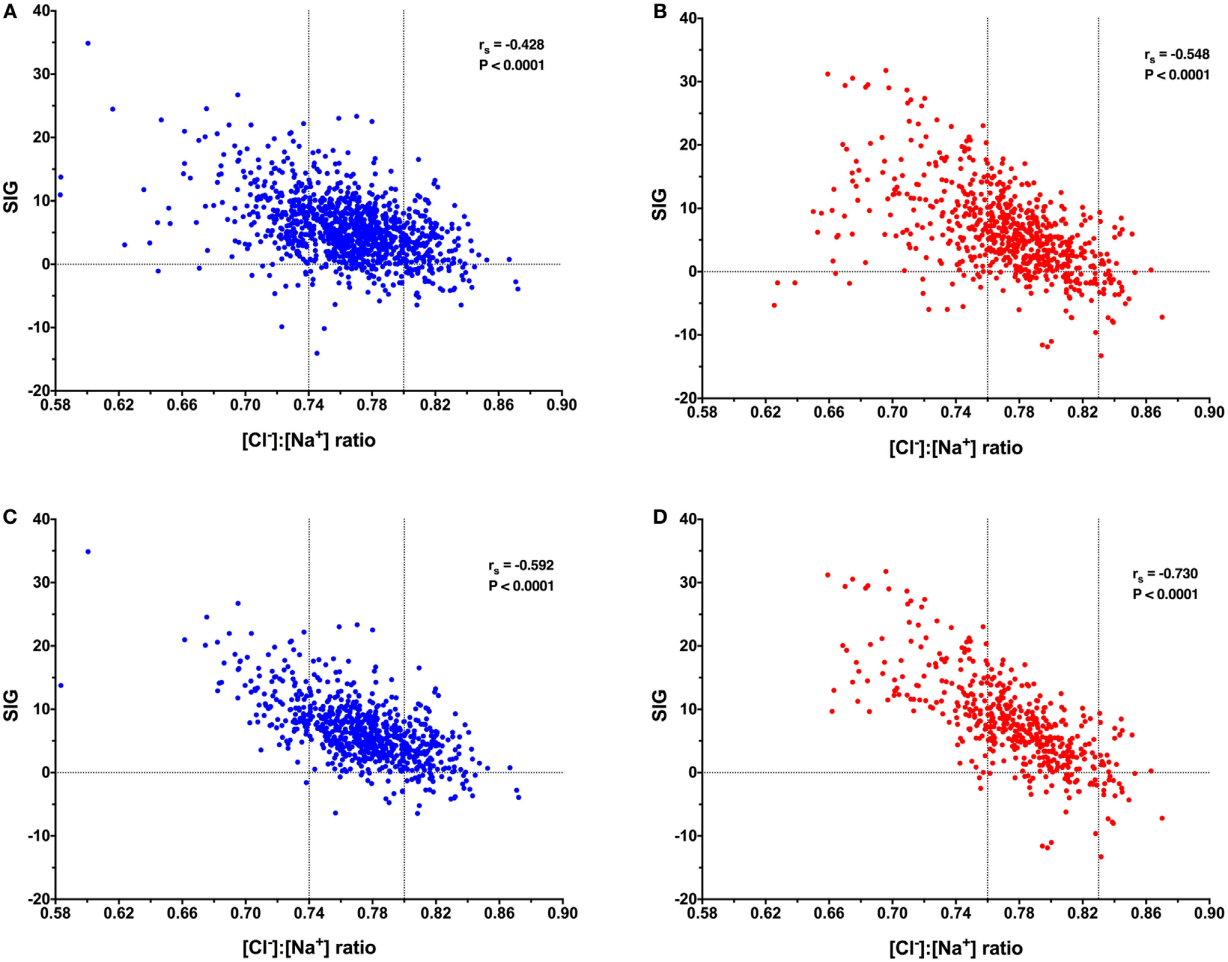
Scatterplots of canine (blue) and feline (red) [Cl^−^]:[Na^+^] ratio values against strong ion gap (SIG) values. Horizontal dotted lines indicate SIG of 0. Vertical dotted lines indicate the site-specific reference values for [Cl^−^]:[Na^+^] ratio. **(A)** Data from 1,106 canine blood gas and electrolyte analyses suggest a significant moderate negative correlation between [Cl^−^]:[Na^+^] ratio and SIG, Spearman’s rho (*r*_s_) −0.428, *P* < 0.0001. **(B)** Data from 671 feline blood gas and electrolyte analyses suggest a significant moderate negative correlation between [Cl^−^]:[Na^+^] ratio and SIG, *r*_s_ −0.548, *P* < 0.0001. **(C)** Data from 739 canine blood gas and electrolyte analyses in patients with a metabolic acidosis (base deficit greater than 4.0) suggest a significant moderate negative correlation between [Cl^−^]:[Na^+^] ratio and SIG, *r*_s_ −0.592, *P* < 0.0001. **(D)** Data from 490 feline blood gas and electrolyte analyses in patients with a metabolic acidosis (base deficit greater than 4.0) suggest a significant strong negative correlation between [Cl^−^]:[Na^+^] ratio and SIG, *r*_s_ −0.730, *P* < 0.0001.

**Figure 2:**
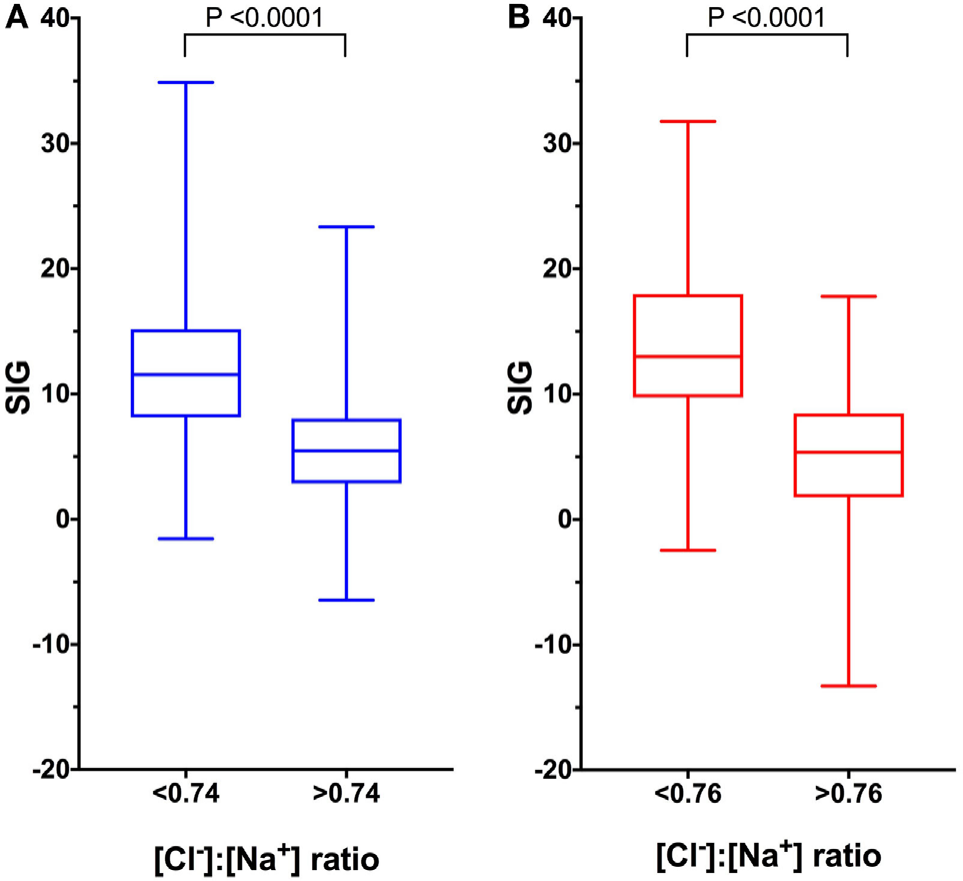
Box and whisker plots of canine (blue) and feline (red) comparing strong ion gap (SIG) values in patients with low and normal or high [Cl^−^]:[Na^+^] ratio values. Boxes represent the 25th and 75th percentiles, the middle line represents the median and the whiskers represent minimum and maximum values. **(A)** In dogs, SIG is significantly greater in animals with metabolic acidosis and hypochloremia as predicted by site-specific reference values for [Cl^−^]:[Na^+^] of < 0.74 than in animals with normal or high [Cl^−^]:[Na^+^] ratios, *P* < 0.0001 by Mann–Whitney *U* test (*n* = 739). **(B)** In cats, SIG is significantly greater in animals with metabolic acidosis and hypochloremia as predicted by site-specific reference values for [Cl^−^]:[Na^+^] of <0.76 than in animals with normal or high [Cl^−^]:[Na^+^] ratios, *P* < 0.0001 by Mann–Whitney *U* test (*n* = 671).

After removal of cases for which requisite data were missing, there were 1,106 records available for acid–base analysis using the quantitative strong ion approach. Using the whole dataset, there was a mild, but significant negative correlation (*r*_s_ = −0.428, *P* < 0.0001) between the [Cl^−^]:[Na^+^] ratio and SIG (Figure 1A). In dogs with a metabolic acidosis (*n* = 739) (defined as BD greater than 4.0) the correlation between the [Cl^−^]:[Na^+^] ratio and the SIG was greater (*r*_s_ = −0.592, *P* < 0.0001) (Figure 1C). The SIG was significantly greater in dogs with a metabolic acidosis in which hypochloremia (defined using the site-specific [Cl^−^]:[Na^+^] ratio < 0.74) was present than in which it was absent ([Cl^−^]:[Na^+^] ratio > 0.74) 11.5 (34.9 to −1.6) with hypochloremia vs. 5.5 (23.3 to −6.5) without hypochloremia (*P* < 0.0001) (Figure 2A).

### Cats

There were 7,604 complete feline electrolyte profiles available for examination. Based on calculations using equation 1, 4,045 profiles had normal chloride, 3,368 profiles were hypochloremic, and 191 profiles showed evidence of hyperchloremia. The previously reported normal ranges for the [Cl^−^]:[Na^+^] ratio (0.74–0.80) correctly identified 3,152 of the normal profiles, 1,276 of the hypochloremic profiles, and 191 of the hyperchloremic profiles for a total accuracy percentage of 60.74%. Using the site-specific RI (0.76–0.83) for [Cl^−^]:[Na^+^] ratio evaluation in cats provided 93.25% accuracy. With the simplified range, 4,042 of the normal profiles were correctly identified, 2,858 of the hypochloremic profiles were correctly identified, and 191 of the hypochloremic profiles were correctly identified. Using the site-specific RI led to a net improvement in accuracy of 32.51% over the previously published RI.

As with dogs, only profiles with normal sodium were evaluated when assessing the performance of the Na-Cl difference RIs. There were 4,472 feline profiles with a normal sodium concentration. Based on the established corrected chloride calculation, of the 4,472 profiles that had normal sodium concentrations, 2,468 profiles had normal chloride, 1,919 profiles were hypochloremic, and 85 profiles showed evidence of hyperchloremia. The currently published normal ranges for the Na-Cl difference (32–40) correctly identified 1,656 of the normal profiles, 618 of the hypochloremic profiles, and 85 of the hyperchloremic profiles for a total accuracy percentage of 52.75%. Using the site-specific RI for Na-Cl difference (26.0–36.0), correctly identified 2,350 of the normal profiles, 1,823 of the hypochloremic profiles, and 78 of the hypochloremic profiles, for a total accuracy percentage of 95.06%. This yielded an overall improvement of 42.31% in accuracy over the previously published RI.

After removal of cases for which requisite data were missing, there were 671 records available for acid–base analysis using the quantitative strong ion approach. Using the whole dataset, there was a moderate, but significant negative correlation (*r*_s_ = −0.548, *P* < 0.0001) between the [Cl^−^]:[Na^+^] ratio and SIG (Figure 1B). In cats with a metabolic acidosis (*n* = 490) (defined as BD greater than 4.0) the correlation between the [Cl^−^]:[Na^+^] ratio and the SIG was greater (*r*_s_ = −0.730, *P* < 0.0001) (Figure 1D). The SIG was significantly greater in cats with a metabolic acidosis in which hypochloremia (defined using the site-specific [Cl^−^]:[Na^+^] ratio < 0.76) was present (*P* < 0.0001) than in which it was absent ([Cl^−^]:[Na^+^] ratio > 0.76) (Figure 2B).

## Discussion

Rapid identification of complex metabolic acid–base disturbances is important in veterinary emergency and critical care practice. The present study suggests that [Cl^−^]:[Na^+^] ratios accurately predict corrected chloride disorders and correlate well with the presence of unmeasured anions, particularly in patients with an increased BD. Data from the present study suggest that the combination of a low [Cl^−^]:[Na^+^] ratio coupled with a BD greater than 4.0 should prompt the clinician to seek the presence of anions such as lactate, ketoacids, salicylates, or ethylene glycol metabolites. A normal [Cl^−^]:[Na^+^] ratio refutes an independent chloride disorder, and in the absence of a metabolic acidosis makes the presence of clinically relevant concentrations of unmeasured anions unlikely. A high [Cl^−^]:[Na^+^] ratio indicates a corrected hyperchloremia is present, and in patients with a metabolic acidosis makes the presence of unmeasured anions less likely.

Using the [Cl^−^]:[Na^+^] ratio approach offers several potential advantages over use of the anion gap ([Na^+^] + [K^+^] − [Cl^−^] − [HCO_3_^−^]) for assessment of metabolic acid–base disturbances. The anion gap calculation incorporates the calculated [HCO_3_^−^] that depends upon the measured CO_2_ partial pressure. Thus, in patients with a marked respiratory acid–base abnormality, the AG calculation may be inaccurate. In contrast, the BD is a CO_2_ independent measure of acid–base status. Additionally, if only the [Cl^−^] and [Na^+^] values are available, such as on a chemistry panel, the ratio may be still be calculated and used to suggest the presence of unmeasured anions and by using both the low and high reference values the ratio may also help exclude the presence of unmeasured anions.

Attempts to improve the identification of the nature, cause, and severity of metabolic acidosis in clinical patients has driven the development of acid–base analysis methods, including the Stewart approach (4), the strong ion concept (15, 16), and the quantitative acid–base analysis systems (17, 18). In contrast to the Henderson–Hasselbalch approach, the strong ion concept posits that disorders of sodium and chloride are two of the major determinants of acid–base disturbances. Hyperchloremia can be readily identified with the [Cl^−^]:[Na^+^] ratio and is associated with a metabolic acidosis. Hypochloremia as a primary disorder causes a metabolic alkalosis. In patients with increased concentrations of negatively charged ion species such as lactate, ketoacids and ethylene glycol metabolites, the presence of hypochloremia may mask or diminish the resulting metabolic acidosis. Measuring these other ion species may be costly and time-consuming. While validated point-of-care assays for lactate and ketones now exist (19–21), they are not universally available and their measurement is unnecessary in every case. Data from the present study suggest that evaluating the BD and the [Cl^−^]:[Na^+^] ratio in combination will identify those patients in which a compensating hypochloremia (10), is present and hence highlight those patients in which additional measurements are warranted. This may be particularly valuable in patients with concurrent respiratory disorders in which the calculated anion gap may be misleading.

Data from the present study also suggest that site-specific reference values for the [Cl^−^]:[Na^+^] ratio and the [Na^+^] − [Cl^−^] difference more accurately predict corrected chloride disorders than do previously published values. Differences in reference values for these parameters between institutions may lead to misinterpretation of important patient data with potential diagnostic or therapeutic consequences. It should be noted that site-specific reference values for both the [Cl^−^]:[Na^+^] ratio and the [Na^+^] − [Cl^−^] difference are best used as clinical heuristics, rather than true RIs. The [Cl^−^]:[Na^+^] ratio and the [Na^+^] − [Cl^−^] difference may offer a “shortcut” to help clinicians to rapidly identify patients with chloride or mixed acid–base disorders such that closer attention may be focused where necessary.

The present study suggests that calculating site-specific reference values improves the accuracy of the [Cl^−^]:[Na^+^] ratio and the [Na^+^] − [Cl^−^] difference compared to those reported previously. Recently, canine RIs for a wide range of acid–base and electrolyte measures were published, based on evaluations of 246 dogs (8). That study suggested wider RIs for a number of parameters than had been previously reported. Of particular relevance here, that study suggested a [Cl^−^]:[Na^+^] ratio RI of 0.71–0.80. The reference values for [Na^+^] and [Cl^−^] in that study were 146.5 (139.3–154.5) and 111.0 ± 3.1, respectively. If these values are used to calculate site-specific reference values via equations 2 and 3 above then values of 0.715–0.800 are obtained. These almost exactly match those reported by Vanova-Uhrikov et al. (8) and suggest that equations 2 and 3 can be correctly applied to other populations. That study also reported a [Na^+^] − [Cl^−^] difference RI of 28.9–43.3. Using equations 4 and 5 to calculate site-specific reference values for that study results in values of 29.3–41.7, which are also quite close to the RIs for [Na^+^] − [Cl^−^] difference reported by Vanova-Uhrikov and others.

In summary, the present study suggests that in order for [Cl^−^]:[Na^+^] ratio and [Na^+^] − [Cl^−^] difference to accurately identify corrected chloride disorders, site-specific reference values for these parameters are required. The present study also suggests that the [Cl^−^]:[Na^+^] ratio is a useful guide for the identification of the presence of unmeasured anions in both dogs and cats particularly where only these values are available or with coexistant respiratory acid–base disturbances. Further work will be required to determine if site-specific reference values for [Cl^−^]:[Na^+^] ratio and [Na^+^] − [Cl^−^] difference improve detection rates for acid–base disturbances in clinical practice and offer opportunities to improve patient outcomes.

## Ethics Statement

The study was exempt from ethics committee approval because it presents a retrospective analysis of electrolyte and acid–base data collected as part of clinician-driven care provided to patients treated at the institution hospital. No client or patient identifying information is presented.

## Author Contributions

RG conceived the study, analyzed data, and cowrote the manuscript; MM analyzed data and cowrote the manuscript; EZ analyzed data and edited the manuscript; SR and DF collected and analyzed data and edited the manuscript.

## Conflict of Interest Statement

The authors declare that the research was conducted in the absence of any commercial or financial relationships that could be construed as a potential conflict of interest.
